# MicroRNA dysregulation and esophageal cancer development depend on the extent of zinc dietary deficiency

**DOI:** 10.18632/oncotarget.7561

**Published:** 2016-02-21

**Authors:** Louise Y. Fong, Cristian Taccioli, Ruiyan Jing, Karl J. Smalley, Hansjuerg Alder, Yubao Jiang, Paolo Fadda, John L. Farber, Carlo M. Croce

**Affiliations:** ^1^ Department of Pathology, Anatomy and Cell Biology, Thomas Jefferson University, Philadelphia, PA, USA; ^2^ Sidney Kimmel Cancer Center, Thomas Jefferson University, Philadelphia, PA, USA; ^3^ Animal Medicine, Production and Health Department, University of Padua, Padua, Italy; ^4^ Department of Molecular Virology, Immunology, and Medical Genetics, Comprehensive Cancer Center, The Ohio State University, Columbus, OH, USA

**Keywords:** microRNA expression profiling, esophageal squamous cell carcinoma, dietary zinc deficiency dose-response, miR-223, miR-21

## Abstract

Zinc deficiency (ZD) increases the risk of esophageal squamous cell carcinoma (ESCC), and marginal ZD is prevalent in humans. In rats, marked-ZD (3 mg Zn/kg diet) induces a proliferative esophagus with a 5-microRNA signature (miR-31, -223, -21, -146b, -146a) and promotes ESCC. Here we report that moderate and mild-ZD (6 and 12 mg Zn/kg diet) also induced esophageal hyperplasia, albeit less pronounced than induced by marked-ZD, with a 2-microRNA signature (miR-31, -146a). On exposure to an environmental carcinogen, ∼16% of moderate/mild-ZD rats developed ESCC, a cancer incidence significantly greater than for Zn-sufficient rats (0%) (*P* ≤ 0.05), but lower than marked-ZD rats (68%) (*P <* 0.001). Importantly, the high ESCC, marked-ZD esophagus had a 15-microRNA signature, resembling the human ESCC miRNAome, with miR-223, miR-21, and miR-31 as the top-up-regulated species. This signature discriminated it from the low ESCC, moderate/mild-ZD esophagus, with a 2-microRNA signature (miR-31, miR-223). Additionally, *Fbxw7, Pdcd4,* and *Stk40* (tumor-suppressor targets of miR-223, -21, and -31) were downregulated in marked-ZD cohort. Bioinformatics analysis predicted functional relationships of the 3 tumor-suppressors with other cancer-related genes. Thus, microRNA dysregulation and ESCC progression depend on the extent of dietary Zn deficiency. Our findings suggest that even moderate ZD may promote esophageal cancer and dietary Zn has preventive properties against ESCC. Additionally, the deficiency-associated miR-223, miR-21, and miR-31 may be useful therapeutic targets in ESCC.

## INTRODUCTION

Esophageal cancer, including esophageal squamous cell carcinoma (ESCC) and adenocarcinoma, is the eighth most common cancer worldwide and the sixth most common cause of death from cancer, with a 5-year survival rate of only 10%. In 2012, there were an estimated 456,000 new cases and 400,000 deaths [1]. Representing 80% of cases of esophageal cancer worldwide, ESCC is the predominant histological subtype [2]. ESCC is typically diagnosed at an advanced stage because early symptoms are usually absent. Thus, clarification of pathogenesis mechanisms and new methods for prevention, diagnosis, and treatment are urgently needed.

Risk factors for ESCC include alcohol and tobacco use, nutritional deficiencies, and exposure to environmental carcinogens, such as *N*-nitrosomethylbenzylamine (NMBA) [3]. In particular, zinc (Zn) deficiency (ZD) (defined as inadequate dietary Zn intake) is implicated in the etiology of ESCC in many populations [4–9], including people with heavy alcohol consumption [10]. In 2005 Abnet et al. [11] showed that high esophageal tissue Zn concentration was strongly associated with a reduced risk of developing ESCC as compared with low tissue Zn concentration; their data provide the strongest evidence in humans of an association between dietary ZD and ESCC.

Previously, we showed that rats on a low Zn diet containing 3 mg Zn/kg (hereafter called marked-ZD) for 5 weeks develop a hyperplastic esophagus with a distinct gene signature that includes upregulation of the proinflammation mediators *S100a8/a9* [12]. Prolonged ZD (23 weeks) leads to an expanded cancer-associated inflammatory program that, when combined with non-carcinogenic low doses of the environmental carcinogen NMBA, produced ESCC [13]. In addition, prolonged ZD by itself induced an oncogenic microRNA (miRNA) signature with miR-31 as the top upregulated species [14], a feature of human ESCCs as well [15, 16]. MiRNAs are short, non-coding RNAs that regulate gene expression by means of translational inhibition and mRNA degradation [17]. Each miRNA inhibits multiple target genes or entire signaling pathways. Thus, a range of biological processes can be affected, including cell proliferation, differentiation, and apoptosis. By inhibiting a variety of tumor suppressive and oncogenic mRNAs [18, 19], miRNAs can act as oncogenes or tumor suppressors depending on tissue or cell-type [20]. Alterations in the expression of miRNA genes contribute to the pathogenesis of most human malignancies [19, 21], including ESCC [15, 16, 22–35].

Our ZD rat model [12–14, 36–38] recapitulates features of human ESCC, including ZD, miRNA dysregulation, and inflammation [4, 15, 16, 39]. Thus, our model provides an opportunity to better define the relationship between dietary Zn intake and miRNA dysregulation in ESCC development. This ZD model with 3 mg Zn/kg diet is relevant to human health. The recommended dietary allowance (RDA) for Zn in males is 11 mg (NIH ODS). Assuming an adult male human on a 3 mg Zn/kg diet consumes about 1.2 kg (2.64 lb) of food/day, his daily Zn intake would be 3.6 mg Zn or 33% of RDA for Zn. Thus, this person would be considered as markedly-ZD. In rat studies by others [40, 41], a “severely” ZD diet has less than 1 mg Zn/kg, a “marginally” ZD diet has 5 mg Zn/kg, and a “marginally “Zn-adequate diet has 10 mg Zn/kg. To extrapolate this to human Zn nutrition, a person on a 1, 5, and 10 mg Zn/kg experimental diet would have a daily Zn intake of about 11%, 54%, 108% of human RDA.

ZD is recognized as a major worldwide public health problem [42–46], affecting 31% of the global population (4-73%, depending on subregions), with higher rates in developing countries [45]. Whereas severe or clinical ZD is uncommon, mild-to-moderate ZD is prevalent throughout the world [47]. Using a well-characterized ZD rat esophageal cancer model [13, 14, 37, 38], the current study asks whether moderate-ZD (6 mg Zn/kg diet, ∼66% of human RDA) and mild-ZD (12 mg Zn/kg diet, ∼132% of human RDA) might cause alterations in miRNA expression, as does a marked-ZD diet (3 mg Zn/kg, ∼33% of human RDA) that provides a microenvironment conducive to ESCC development on exposure to low carcinogen doses [14]. For this, we conducted a long-term tumor bioassay by low doses of NMBA in rats fed diets with different amounts of Zn - 3, 6, 12, or 60 mg Zn/kg to represent marked-ZD, moderate-ZD, mild-ZD, and Zn-sufficiency (ZS), respectively. In parallel, we performed miRNA profiling (nanoString platform) in esophageal mucosa from NMBA-treated rats at tumor endpoint and from NMBA-untreated rats at identical time point in order to correlate miRNA expression changes with ZD doses and esophageal tumor outcome.

## RESULTS

### Dose-response studies with dietary ZD in esophageal tumorigenesis

**Figure 1:**
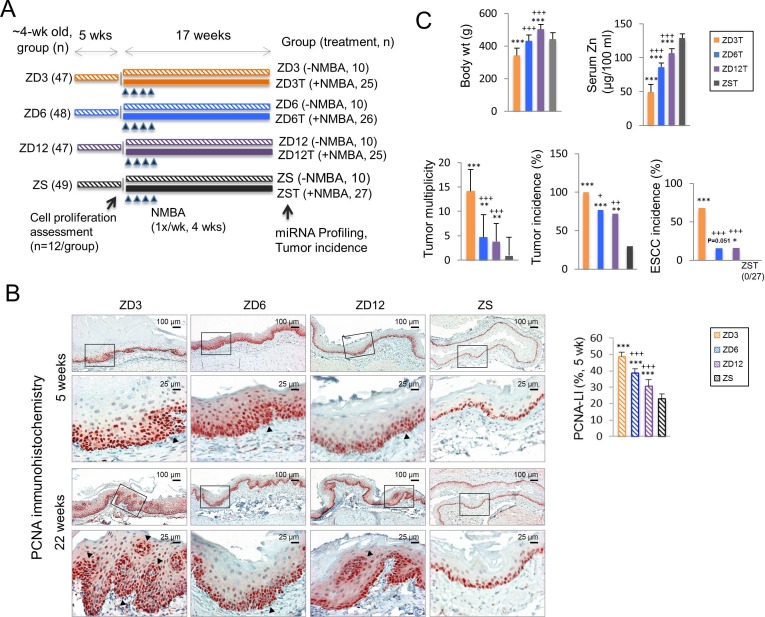
Dose-response relationship between dietary Zn deficiency and esophageal cancer development **A.** Study design - 4-week-old rats were fed diets containing 3, 6, 12, or 60 mg Zn/kg, forming marked-ZD (ZD3), moderate-ZD (ZD6), mild-ZD (ZD-12), and Zn-sufficient (ZS) groups (n = 47-49/group). After 5 weeks, NMBA-treated tumor (T) groups received 4 intragastric NMBA doses, once a week for 4 consecutive weeks (2 mg/kg body weight; *n* = 26-27 rats/group); control groups were NMBA-untreated (*n* = 10 rats/group). The study was concluded 17 weeks after the 1st NMBA dose. **B.** At 5 weeks after dietary regimen, immunohistochemical analysis showing abundant and strong PCNA-positive nuclei (red, 3-amino-9-ethylcarbazole substrate-chromogen) in in multiple cell layers (arrow head) in proliferative ZD3, ZD6, and ZD12 esophageal epithelia *vs* few PCNA-positive nuclei in nonproliferative ZS control (scale bars = 100 μm, x100 magnification). Open rectangles are insets illustrated in panels directly below (scale bars = 25 μm, x400 magnification). PCNA-LI (labeling index, %) was significantly higher in ZD3 *vs* ZS, ZD6 *vs* ZS, ZD12 *vs* ZS group, and significantly higher in ZD3 *vs* ZD6, and ZD3 *vs* ZD12 group (***P < 0.001, ^+++^*P* < 0.001, *n* = 12 rats/group). At 22-weeks, ZD3, ZD6, and ZD12 esophagi showed focal hyperplastic lesions (arrow head) with abundant PCNA-positive nuclei *vs* nonproliferative ZS control (scale bars = 100 μm, x100 magnification). Open rectangles are insets illustrated in panels directly below (scale bars = 25 μm, x400 magnification). **C.** Tumor endpoint (*n* = 26-27 rats/group): body weight (g), serum Zn levels (μg/100 ml), esophageal tumor multiplicity (number of tumors/esophagus), tumor incidence (%), and esophageal squamous cell carcinoma (ESCC) incidence (%), (ZD tumor group *vs* ZST control: ***P < 0.001, **P < 0.01, *P < 0.05. ZD6T *vs* ZD3T and ZD12T *vs* ZD3T: ^+++^*P* < 0.001, ^++^*P* < 0.01, ^+^*P* < 0.05). Error bars represent standard deviation.

To determine whether moderate-ZD (6 mg Zn/kg diet) and mild-ZD (12 mg Zn/kg diet) [40, 41] cause aberrant miRNA expression and enhance NMBA-induced esophageal tumorigenesis, a 22-week tumor study was performed. As shown in the study design (Figure 1A), 191 rats (4-wk-old) were fed diets containing 3, 6, 12, or 60 mg Zn/kg to form respective ZD3T, ZD6T, ZD12T, and ZST (NMBA-treated tumor group, *n* = 25-27 rats/group), and ZD3, ZD6, ZD12, and ZS (diet group without NMBA treatment, *n* = 22 rats/group). Tumor groups received four intragastric doses of NMBA (2 mg/kg body weight, once/week for four consecutive weeks). At endpoint (week 22), miRNA-expression profiling was performed on esophageal mucosa-derived RNA from both diet and tumor groups. At the same time, esophagi from the tumor group were evaluated for tumor incidence.

First, we determined whether after 5 weeks of dietary regimen, moderate and mild-ZD also increased esophageal cellular proliferation, as did marked-ZD [38, 48]. PCNA immunohistochemistry was used to identify cells in S-phase [49]. ZD6 and ZD12 esophageal epithelia displayed abundant PCNA-positive nuclei in several cell layers, including suprabasal layers, although less prominent compared to ZD3 esophagus. By contrast, PCNA-positive nuclei in ZS esophagus were largely restricted to the basal cell layers (Figure 1B, 5-weeks). The cell proliferation index (% of intensely stained PCNA-positive nuclei) in ZD cohorts, namely, ZD3 (49±2.9%), ZD6 (39±2.7%), or ZD12 (31±4%) esophagus, was significantly greater than ZS esophagus (23±2.9%) (*P* < 0.001) (Figure 1B). Among the ZD cohorts, ZD3 showed significantly higher PCNA-labeling index than ZD6 or ZD12 esophagus (*P* < 0.001). At the conclusion of the study (22-weeks), ZS esophagus remained nonproliferative, whereas, ZD6 and ZD12 esophagus showed sustained proliferation with PCNA-positive nucleic in focal hyperplastic lesions (FHLs), a result consistent with the highly proliferative ZD3 esophagus, albeit less pronounced (Figure 1B, 22-weeks). These findings demonstrate that moderate/mild-ZD causes sustained esophageal cellular proliferation.

At tumor endpoint, serum Zn levels in ZD cohorts were significantly lower than in ZS rats (*P* < 0.001) (Figure 1C). Moderate-ZD and ZS rats had comparable body weight, because ZS group was paired-fed to moderate-ZD rats to match their relative reduced food intake (Figure 1C). Moderate/mild-ZD rats also had higher body weight than marked-ZD rats, because of reduced food consumption in the latter group.

**Figure 2:**
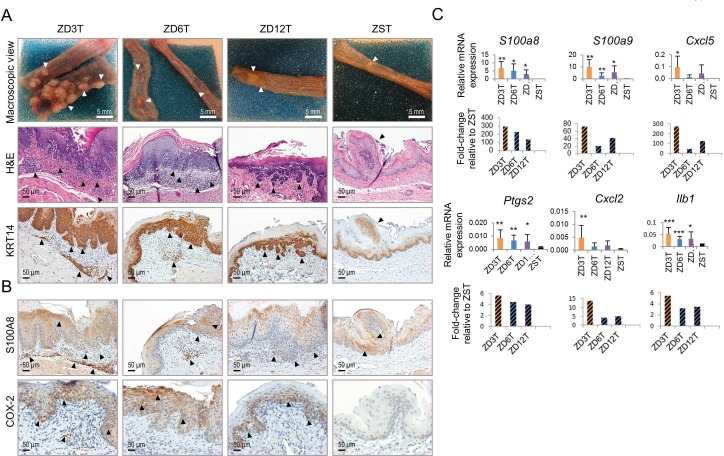
Esophageal tumor development in marked-ZD, moderate-ZD, and mild-ZD rats **A.** Macroscopic view of representative esophagus from a ZD3T rat showing multiple tumors, ZD6T and ZD12T rats showing sessile tumors, and from a ZST showing an occasional papilloma (arrow heads); representative hematoxylin & eosin (H&E)-stained esophageal sections and near serial KRT14 (squamous cell tumor marker) immuno-stained sections (brown, 3,3′-diaminobenzidine tetrahydrochloride) showing invasive ESCC (arrow head) in ZD3T, ZD6T, and ZD12T esophagi, and a papilloma (arrow) in ZST esophagus. Scale bars = 50 μm, x200 magnification. **B.** Inflammation marker S100A8 and COX-2 showed strong cytoplasmic staining (brown, 3,3′-diaminobenzidine tetrahydrochloride) in hyperplastic epithelia and tumor area in ZD3T, ZD6T, and ZD12T esophagus. Scale bars = 50 μm, x200 magnification. **C.** qPCR analysis of mRNA expression of six selected inflammation genes *S100a8, S100a9, Cxcl5*, *Ptgs2, Cxcl2,* and *Ilb1* (ZD3T *vs* ZST, ZD6T *vs* ZST, or ZD12T *vs* ZST group: ***P < 0.001, **P < 0.01, *P < 0.05) (n = 7-10 rats/group, Oaz1 as normalizer, and error bars represent standard deviation). ZD3T, ZD6T, ZD12T, and ZST represent, respectively, marked-ZD, moderate-ZD, mild-ZD, and Zn-sufficient tumor groups.

Regardless of the extent of the deficiency of Zn, ZD cohorts showed significantly higher tumor incidence and multiplicity than ZS rats (Figure 1C; Figure 2A, macroscopic view). Moderate and mild-ZD groups had similar esophageal tumor incidence (∼72%) and multiplicity (∼4 tumors/esophagus), results that were significantly lower than those in marked-ZD (100%, 14±4.5) but greater than ZS rats (29.6%, 0.92±3.8) (*P* < 0.001). Histological examination (H&E-stained sections), as well as KRT14 immunostaining (biomarker for ESCC [50–52]), showed that 68% (17/25) of ZD3T, 15.4% (4/26) of ZD6, and 16% (4/25) of ZD12 rats harbored ESCC. Nutritionally complete ZST rats did not develop cancer (0/27). All tumors in ZST rats were papillomas [13, 53]. The difference in ESCC incidence between ZD12T and ZST groups was statistically significant (ZD12T vs ZST, *P* = 0.047) and that between ZD6T vs ZST group was close to statistical significance (*P* = 0.051). These data established for the first time that mild and moderate-ZD enhances esophageal tumorigenesis and promotes progression to ESCC.

### Heightened Inflammation accompanies increased esophageal tumorigenesis in moderate and mild-ZD rats

Previously, we reported that marked-ZD (3 mg Zn/kg diet) causes upregulation of numerous cancer-associated inflammation genes that fuel ESCC progression [13]. Whether moderate and mild-ZD also causes chronic inflammation in the esophagus was evaluated by analyzing the expression of six cancer-associated inflammation genes (*S100a8*, *S100a9*, *Cxcl5*, *Ptgs2, Cxcl2,* and *Ilb1*) [13] using quantitative polymerase chain reaction (qPCR). Consistent with our previous study [13], the high tumor-burden, ZD3T esophagus showed upregulation of all six inflammation genes compared to its ZST counterpart (Figure 2C). Importantly, ZD6T and ZD12T esophagus also showed statistically significant upregulation of four inflammation genes - *S100a8, S100a9*, *Ptgs2*, and *Ilb1* (ZD6T vs ZST, ZD12T vs ZST, *P* < 0.05 to *P* < 0.001, Figure 2C). Immunohistochemical analyses in ZD6T and ZD12T esophagus showed that the upregulation observed at the transcript levels for *S100a8* and *Ptgs2* was also reflected at the protein level (Figure 2B, S100A8 and COX-2*)*. These data establish that moderate/mild-ZD up-regulates key inflammation genes in esophageal tumourigenesis [54, 55].

### MiRNA expression profiles distinguish the extent of ZD in esophagus

**Figure 3:**
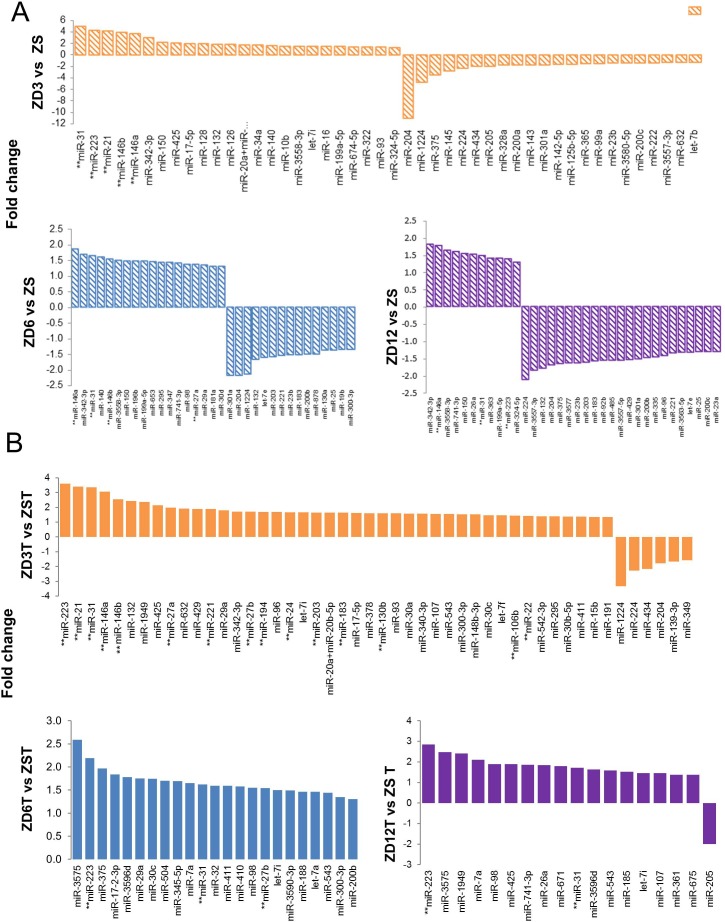
Esophageal microRNA expression profiling in Zn modulated rats by nanoString™ nCounter rat miRNA expression assay kit **A.**–**B.** Barplots showing fold change of differentially expressed miRNAs in A. NMBA-untreated control esophagi at 22-weeks after dietary regimen: ZD3 *vs* ZS, ZD6 *vs* ZS, and ZD12 *vs* ZS group; and B. Tumor bearing esophagi from NMBA-treated rats at tumor endpoint: ZD3T *vs* ZST, ZD6T *vs* ZST, and ZD12T *vs* ZST. **Denotes miRNAs that are similarly up- or down-regulated in human esophageal squamous cell carcinoma. ZD3 = marked-ZD; ZD6 = moderate-ZD; ZD12 = mild-ZD; ZS = Zn-sufficient. ZD3T, ZD6T, ZD12T, and ZST represent the tumor groups (*n* = 6 rats/group; cut off: *P* ≤ 0.05, fold-change ≥ 1.3).

To determine the extent of differential miRNA expression in esophagus across different doses of dietary ZD, we performed miRNA expression profiling in ZD3, ZD6, ZD12, and ZS esophageal mucosa using the nanoString nCounter rat miRNA expression assay kit (*n* = 6 rats/group) (nanoString Technologies, Seattle, WA). This assay detects 423 rat miRNAs. The nanoString platform directly measures miRNA expression levels without reverse transcription or PCR amplification, thereby eliminating enzymatic bias [56, 57]. ZD esophageal miRNA profiles, including for ZD3, ZD6, and ZD12 cohorts, were different from ZS esophageal miRNA profile (Figure 3A). Using a cutoff point of *P* < 0.05 and >1.3-fold difference, we identified 46 dysregulated miRNAs in ZD3 esophagus (24 up- and 22 down-regulated), 33 dysregulated miRNAs in ZD6 (18 up- and 15 down-regulated), and 34 dysregulated miRNAs in the ZD12 esophagus (11 up- and 23 down-regulated) (Figure 3A, Supplementary Table 1). Many of the dysregulated miRNAs are similarly dysregulated in human ESCC (Figure 3A, marked by asterisks). Thus, ZD3 esophagus displayed a 5-miRNA signature resembling the miRNAome of human ESCC. The signature was defined by five top up-regulated oncogenic miRNAs (miR-31, -223, -21, -146b, -146a) [15, 16, 22–26, 29, 30, 34, 58] that were up 4.9-3.7 fold. This result is consistent with our previous study that employed a mouse miRNA expression assay kit to profile rat esophagus [14]. Similarly, ZD6 esophagus had a 4-miRNA signature (miR-146a, -31, -146b, -27a; up 1.9-1.4 fold) and ZD12 esophagus a 3-miRNA signature (miR-146a, -31, -223; up 1.8-1.4 fold). Notably, miR-31 and miR-146a were differentially expressed in ZD6/ZD12 esophagus as they were in ZD3 esophagus, albeit at a lower expression level. The oncomiR miR-21 that was prominently overexpressed (up 4.2 fold) in the highly hyperplastic ZD3 esophagus, however, was not differentially expressed in the less hyperplastic ZD6 or ZD12 esophagus (Figure 1B: PCNA, 22 weeks). These findings show that moderate and mild-ZD induces alterations in miRNA expression, including miR-31 and miR-146a. Additionally, miRNA signatures distinguish the highly hyperplastic esophageal phenotype induced by marked-ZD from the less hyperplastic esophageal phenotype induced by moderate and mild-ZD.

### MiRNA expression profiles distinguish esophageal tumor progression in rats fed different amounts of Zn

**Figure 4:**
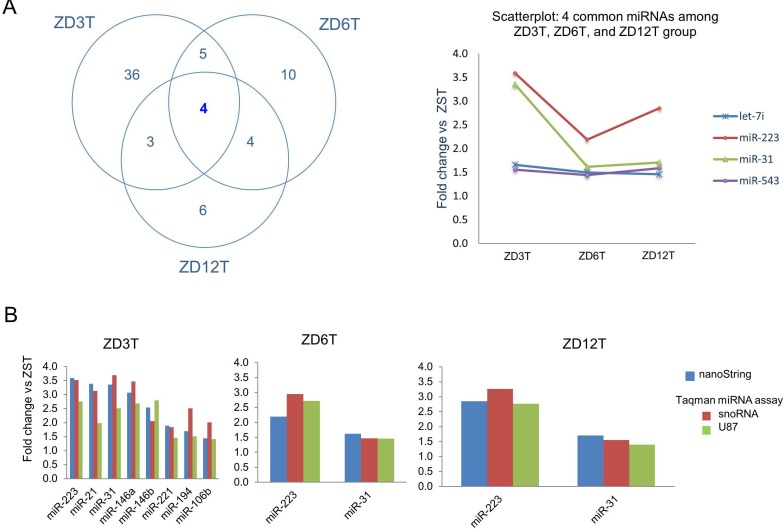
Validation of nanoString miRNA profiling data in Zn-deficient tumor groups by Taqman miRNA assays **A.** Venn diagram showing miR-223 and miR-31 are common to ZD3T, ZD6T, and ZD12T esophagi (cutoff point of *P* < 0.05 and fold difference >1.3), and scatterplot showing their fold change *vs* ZST. **B.** Validation of eight representative miRNAs in ZD3T esophagus; and miR-223 and miR-31 in ZD6T and ZD12T esophagi. Quantitative polymerase chain reaction (qPCR) analysis was performed using snoRNA and U87 as normalizers (*n* = 7-10 rats/group). ZD3T, ZD6T, ZD12T, and ZST represent, respectively, marked-ZD, moderate-ZD, mild-ZD, and Zn-sufficient tumor groups.

We next compared the miRNA profiles of tumor-bearing esophagus across different doses of dietary Zn using the nanoString rat miRNA expression assay kit (*n* = 6 rats/group). ZD tumor miRNA profiles (ZD3T, ZD6T, and ZD12T) were distinctly different from ZS tumor (ZST) profile. Using a cutoff point of *P* < 0.05 and >1.3-fold difference, 48 miRNAs were found to be differentially expressed in ZD3T esophagus (42 up- and 6 down-regulated), 23 dysregulated miRNAs in ZD6T esophagus (all up-regulated), and 18 in ZD12T esophagus (17 up- and 1 down-regulated) (Figure 3B, Supplementary Table 2). Importantly, the high ESCC-burden, ZD3T esophagus had a 15-miRNA signature that resembled the human ESCC miRNAome [15, 16, 22, 24–35, 58, 59]. This 15-miRNA signature (Figure 3B, marked by asterisks) was defined by strong to modest upregulation of oncogenic miR-223, -21, -31, -146a, -146b, -27a, -221, -27b, -194, -24, -203, -183, -130b, -106b, and -22 (up 3.6 to 1.4 fold). By contrast, low ESCC-burden, ZD6T and ZD12T esophagus displayed, respectively, a 3-miRNA signature (miR-223, -31, -27b) and a 2-miRNA signature (miR-223, -31; up 2.9 and 1.5 fold) with modest upregulation (Figure 3B). A Venn diagram (Figure 4A) showed that miR-31, -223, -7i, -543 were the four common miRNAs shared among ZD3T, ZD6T and ZD12T esophagus. Among which, miR-31 [15, 16, 30, 60] and miR-223 [25, 26, 34] are oncomiRs for human ESCC. Thus, moderate and mild-ZD induces alterations in miRNA expression, including miR-31 and miR-223. In addition, our data show that miRNA-signatures can distinguish the divergent ESCC progression in marked-ZD *vs* moderate/mild-ZD rat cohorts.

### Validation of nanoString miRNA expression profiling data

To validate the nanoString miRNA results, we performed Taqman miRNA expression assays using qPCR and two normalizers - snoRNA and U87 (*n* = 7-10 rats/group). We selected 8 miRNAs in ZD3T esophageal tissue (miR-223, -21, -31, -146a, -146b, -221, -194, and -106b) and two miRNAs (miR-31, -223) in ZD6T and ZD12T esophageal tissues, Figure 4B shows that the Taqman data confirmed the upregulation of all 8 selected miRNAs in ZD3T vs ZST samples, and the upregulation of miR-223 and miR-31 in ZD6T and ZD12T samples. The same fold changes response to dietary ZD was observed using Taqman as with the nanoString platform.

### Cellular localization of miR-223, miR-31 and miR-21 expression in human ESCC tissue

**Figure 5:**
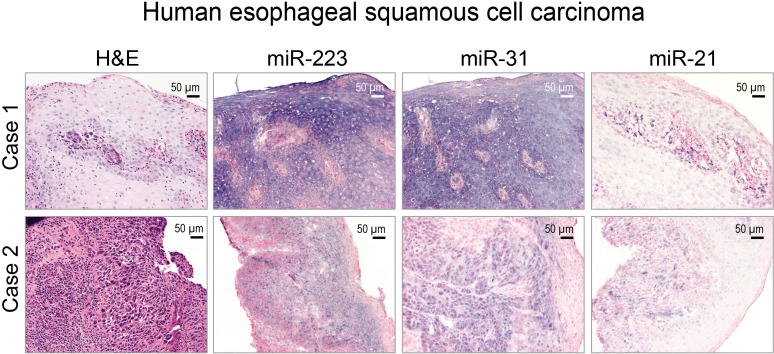
Localization of miR-223, miR-31, and miR-21 in human esophageal squamous cell carcinoma (ESCC) tissue by in situ hybridization (ISH) Representative hematoxylin and eosin [H&E]-stained sections of ESCC tissues (2 cases) are shown. miR-223, -31, and -21 ISH signal (blue, 4-nitro-blue tetrazolium and 5-brom-4-chloro-3′-indolylphosphate; counterstain, nuclear fast red) was moderate to intense and abundant in near serial formalin-fixed, paraffin-embedded sections of ESCC tumor tissue. Scale bars = 50 μm, x200 magnification.

The cellular origins of miRNAs are of importance to their mechanistic roles in cancer development. Previously, we demonstrated an abundant miR-31 ISH signal in human ESCC tissue [60]. Whether miR-223 and miR-21 co-localize in the same ESCC tissue is not known. Thus, we evaluated the cellular localization of all three miRNAs in archived FFPE human ESCC tissues using in situ hybridization (ISH) (*n* = 12 cases). All 12 cases showed intense to moderate miR-31, miR-223, and miR-21 ISH signal in near serial sections of moderately to poorly differentiated ESCC tumor samples (Figure 5). By contrast, the normal mucosa adjacent to the tumor cells had very weak staining (data not shown). These results represent the first simultaneous *in situ* detection of miR-223, -21, and -31 in human ESCC.

### miR-223, -31, -21 upregulation correlates with down-regulation of their tumor-suppressor targets

**Figure 6:**
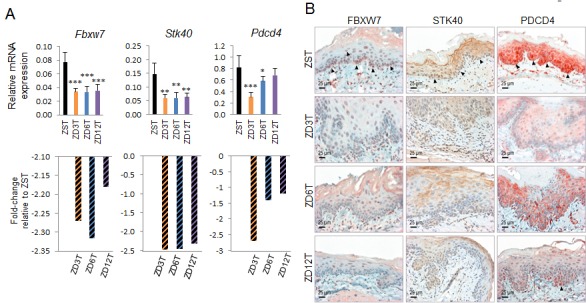
Analysis of esophageal expression of Fbxw7, Stk40, and Pdcd4 (respective tumor suppressor targets of miR-223, miR-31, and -21) in Zn-modulated rats at tumor endpoint **A.** Quantitative polymerase chain reaction (qPCR) analysis of esophageal *Fbxw7*, *Stk40*, and *Pdcd4* expression in three ZD tumor groups (*n* = 7-10 rats/group; Oaz1 as normalizer). *Fbxw7* and Stk40 expression was significantly downregulated in ZD3T, ZD6T, and ZD12T group *vs* ZST group. *Pdcd4* expression was significantly downregulated in ZD3T and ZD6T group vs ZST group. ***P < 0.001, **P < 0.01, *P < 0.05. Error bars represent standard deviation. **B.** Immunohistochemical (IHC) analysis of FBXW7, STK40, and PDCD4 protein expression. IHC showing reduction of FBXW7 and STK40 protein expression (nuclear, cytoplasmic) in ZD3T, ZD6T, and ZD12T esophagus, compared to ZST esophagus (brown, 3,3′-diaminobenzidine tetrahydrochloride). Nuclear PDCD4 protein expression was largely absent in ZD3T esophagus and greatly reduced in ZD6T and ZD12T compared to ZST (red, 3-amino-9-ethylcarbazole substrate-chromogen). ZD3T, ZD6T, ZD12T, and ZST represent, respectively, marked-ZD, moderate-ZD, mild-ZD, and Zn-sufficient tumor groups. Scale bars = 25 μm, x400 magnification.

We then determined whether upregulation of oncogenic miR-223, -31, and -21 in ZD3T, ZD6T, and ZD12T esophagus is accompanied by down-regulation of their respective tumor suppressor targets, *FXBW7* [25, 61], *STK40* [60, 62, 63], and *PDCD4* [64], by using qPCR (*n* = 7-10 rats/group). The mRNA levels of *Fbxw7* and *Stk40* in all 3 ZD tumor groups were statistically significantly reduced as compared to ZST esophagus (**P < 0.01, ***P < 0.001). *Pdcd4* was significantly reduced only in ZD3T and ZD6T esophagus *vs* its ZST counterpart (*P < 0.05, ***P < 0.001) (Figure 6A). Immunohistochemical analysis showed that FBXW7, STK40, and PDCD4 protein was moderately/strongly expressed in the basal/suprabasal cells of ZST esophagus, but reduced or absent in hyperplastic/tumor-bearing ZD3T, ZD6T, and ZD12T esophagus (Figure 6B). Thus, upregulation of oncogenic miR-223, -31, and -21 is accompanied by down-regulation of their respective tumor suppressor target *Fbxw7*, *Stk40* and *Pdcd4*.

### Esophagus-specific functional relationship prediction among tumor-suppressor targets

**Figure 7:**
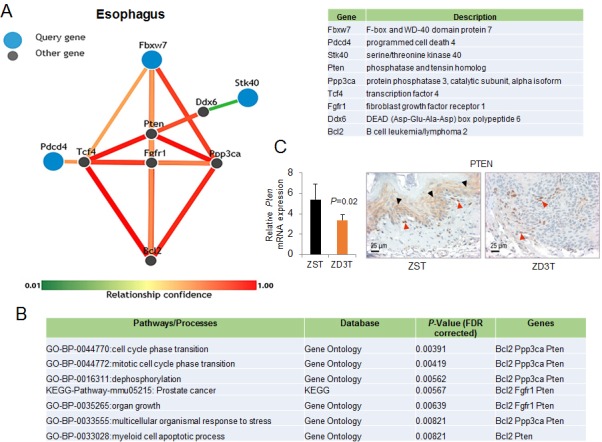
Esophagus-specific functional relationship network among Fbxw7, Stk40, and Pdcd4 using the Functional Networks of Tissues in Mouse (FNTM) prediction tool **A.** The displayed esophagus-specific nine-gene network shows predicted functional relationships among the genes that are most functionally related to *Stk40, Pdcd4 and Fbxw7* (tumor-suppressor targets of miR-31, mir-21 and miR-223, respectively). The edges between genes are colored by the confidence of the predicted relationship. **B.** Enrichment Gene Ontology analysis for the nine genes retrieved from FNTM analysis. Terms exceeding an FDR-corrected *P*-value of 0.01 are excluded. **C.** Quantitative polymerase chain reaction (qPCR) analysis of esophageal *Pten* expression in ZD3T and ZST esophagus (*n* = 6 rats/group; Oaz1 as normalizer, *P = 0.02). Error bars represent standard deviation. **D.** Immunohistochemical analysis of PTEN protein expression in ZD3T and ZST esophagus showing cytoplasmic PTEN expression in ZST esophageal epithelial cells (black arrow head, brown, 3,3′-diaminobenzidine tetrahydrochloride) and lack of PTEN expression in ZS3T esophageal ESCC tumor cells Stromal cells and blood vessels showed intensely positive expression for PTEN (red arrow head, served as integral internal positive). ZD3T and ZST = marked-ZD and Zn-sufficient tumor groups. Scale bars = 25 μm, x400 magnification.

Functional Networks of Tissues in Mouse (FMTN) [65, 66] is a prediction tool for tissue-specific protein interactions for the mouse that is based on the integration of a variety of genomic data and prior knowledge of gene function. To explore the esophagus-specific functional relationships for *Fbxw7, Stk40*, and *Pdcd4* (tumor-suppressor targets of miR-223, -31, -21), we employed FNTM for the rat. The percentage of orthologous genes shared by mouse and rat is very high, and a similar tool is not available for the rat. We obtained a nine-gene network of functional relationship predictions in the esophagus (Figure 7A) showing that *Fbxw7, Stk40*, and *Pdcd4* were functionally related to several cancer-related genes, such as *Pten,* tumor suppressor of miR-21 [67, 68], oncogene *Bcl2* [69], a Wnt signaling pathway transcription factor *Tcf4* [70], the dead box protein family of RNA helicases *Ddx6* [71, 72], fibroblast growth factor receptor 1 *Fgfr1* [73–75], and *Ppp3ca,* a component of calcium/calcineurin signaling that includes apoptosis [76]. Enrichment Gene Ontology analysis showed that *Pten, Bcl2*, *Ppp3Ca,* and *Fgfr1* were the genes most functionally related to *Fbxw7, Stk40*, and *Pdcd4* and were statistically significantly enriched in biological processes related to cell cycle, growth, response to stress, and apoptosis regulation (Figure 7B). We then showed that the expression of the most functionally related gene, *Pten,* was also down-regulated in the ESCC-bearing ZD3T esophagus that overexpressed miR-21 at the mRNA level by qPCR (*P* = 0.02, *n* = 6 rats/group) and at the protein level by immunohistochemistry compared to ZS counterpart (Figure 7C). That the three tumor suppressor targets are predicted to interact to alter network of cancer-related proteins [65, 66, 77] provide support that miR-223, miR-21, and miR-31 have an important role in ESCC and may be useful therapeutic targets in ESCC.

## DISCUSSION

In humans, low dietary Zn intake is associated with an increased risk of ESCC [7, 9]. Although marginal ZD is prevalent in humans [47], the effect of moderate ZD on the etiology of ESCC has not been studied. Using a well-characterized ZD rat esophageal cancer model [13, 14, 37, 38], the current study demonstrates for the first time that ESCC initiation and progression, as well as miRNA dysregulation, depend on the extent of deficiency of dietary Zn.

Sustained increased cellular proliferation is a hallmark of cancer [78]. Previously, we reported that marked-ZD (3 mg Zn/kg) induces prominent esophageal cellular proliferation, predisposing to tumor development [13, 79]. We now show that a moderately-ZD (6 mg Zn/kg) and a mildly-ZD (12 mg Zn/kg) diet also induced a sustained but less-pronounced, esophageal cellular proliferation. Importantly, in the presence of moderate or mild-ZD, low NMBA doses elicited statistically significantly higher tumor incidence/multiplicity, as well as ESCC progression than with Zn sufficiency (Figure 1C). Although marked-ZD led to significantly higher tumor/ESCC incidence than moderate or mild-ZD, no statistically significant difference was obtained in tumor or ESCC outcome between moderate-ZD and mild-ZD, despite a two-fold difference in Zn content. These data show a dose-response relationship between the extent of ZD and ESCC development. Additionally, they provide the first evidence that moderate to mild-ZD, combined with low doses of the environmental carcinogen NMBA, produces ESCC.

MiRNA-expression profiling of human tumors has identified signatures associated with staging, progression, prognosis, and response to treatment [21]. Also, miRNA expression patterns have been shown to be potential classifiers for ESCC [29]. Using the nanoString platform, miRNA expression profiles distinguished the highly preneoplastic/proliferative marked-ZD esophageal phenotype with a 5-miRNA signature (miR-31, -223, -21, -146b, -146a), from the less proliferative, mild-ZD phenotype with a 3-miRNA signature (miR-146a, -31, -223). Importantly, the high ESCC-burden, marked-ZD esophagus showed a 15-miRNA signature (with miR-223, -21, and -31 as the top-up-regulated species), thus differentiating it from the low ESCC-burden, mild-ZD esophagus with a 2-miRNA signature (miR-223, -31). In addition, our data show that these miRNA signatures not only differentiate esophageal preneoplasia from normal esophagus and stages of ESCC progression, but also highlight the molecular impact of dietary ZD on miRNA dysregulation in the pathogenesis of ESCC.

miR-223, miR-21, and miR-31 are the top-upregulated species in the high ESCC-burden, marked-ZD esophagus. In addition, miR-223 and miR-31 dysregulation is common to marked-ZD and moderate/mild-ZD tumor groups (Figure 4A). Recently, we demonstrated by ChIP-seq analysis that in ZD esophagus, the miR-31 promoter region and NF-κB binding site were activated, unleashing miR-31-associated STK40-NF-κB controlled inflammatory signaling to produce a preneoplastic phenotype; Zn-replenishment restores the normal regulation of this genomic region and a normal esophageal phenotype [60]. The mechanism(s) by which miR-223 and miR-21 are upregulated by ZD remains to be elucidated.

miR-223, miR-21, and miR-31 can target many important tumor suppressor genes, including *FXBW7* [25, 61], *STK40* [60, 62, 63], and *PDCD4* [64]. miR-223 acts as an oncomiR in several solid tumors, including ESCC, gastric, ovarian, and bladder cancers [25, 80–82]. In ESCC, patients with high miR-223 expression have a significantly poorer prognosis, presumably because of repression of the function of its tumor suppressor target FBXW7 [25]. FBXW7 is a cell cycle protein that regulates the stability of several oncoproteins, including cyclin E, c-Myc, and c-Jun [83]. miR-21 is one of the most consistently overexpressed oncomiRs in solid cancers [84], including ESCC [22–24, 29]. A genuine oncogene, mice conditionally overexpressing miR-21 develop lymphoma [85]. Oncogenic miR-21 exerts its anti-apoptotic effects by targeting the tumor suppressors PDCD4 and PTEN [64, 67]. PDCD4 is one of the most frequently down-regulated proteins in esophageal cancer [86]. miR-31 acts as an oncomiR in squamous cell carcinomas (SCCs), including ESCC [15, 16], tongue SCC [87], head and neck SCC [88], and skin SCC [89]. *STK40* is a known negative regulator of NF-κB mediated transcription [90] and a miR-31 direct target [60, 62, 63]. That the tumor suppressor genes *Fxbw7*, *Pdcd4*, and *Stk40* were downregulated at the mRNA and protein level in marked-ZD tumor group (Figure 6) and that they were predicted to interact to alter network of target proteins [65, 66, 77] (Figure 7) provide support that miR-223, miR-21, and miR-31 have an important role in ESCC and may be useful prognostic biomarkers and therapeutic targets for ESCC.

A limitation of this study is the fact that the underlying biological mechanisms of the key dysregulated miRNAs in ESCC development, namely, miR-223, miR-21, and miR-31, were not investigated. Studies are in progress to specifically address this issue.

In summary, this dose-response study demonstrates that ESCC development and the underlying miRNA dysregulation are dependent on the extent of deficiency of the nutrient Zn. Although it remains to be determined if the results in this ZD dose-response study in the rat will translate to human ESCC, our findings suggest that dietary Zn may have preventive properties against ESCC and provide a mechanistic rationale for exploring the therapeutic use of Zn against ESCC. In addition, our study has identified Zn deficiency-associated miRNA signatures that may underlie the molecular pathogenesis of ESCC in Zn-deficient populations. Our study suggests that miR-223, miR-31 and miR-21 alone or in combination could be used as therapeutic targets for treatment of ESCC.

## MATERIALS AND METHODS

### Animals, Zn-adjusted diets, and carcinogen

Male weanling Sprague-Dawley rats (50 ± 5 g) were obtained from Taconic Laboratory (Germantown, NY). Four egg white-based, Zn-adjusted diets, hereafter called ZD3, ZD6, ZD12, and ZS, containing 3, 6, 12, and 60. respectively, (mg Zn/kg diet), were from Harlan Teklad (Madison, WI). The diets were shape and color-coded to ensure the animals were fed the assigned food. NMBA was from Midwest Research Institute (Kansas City, MO).

### Experimental design

Animal protocols were approved by the Thomas Jefferson University Institutional Animal Care and Use Committee. Weanling male rats were randomly divided into 4 dietary groups (ZD3, ZD6, ZD12, and ZS, *n* = 47-49 rats/group) and were tail-tattooed for identification. ZD rats were fed ad libitum and ZS rats were pair-fed to ZD6 animals to match the decreased food consumption of ZD6 rats [38]. After 5 weeks, 12 rats per group were killed for evaluation of esophageal cell proliferation [38]. The remaining animals were divided into NMBA-treated groups (*n* = 25-27 rats/dietary group) and NMBA-untreated groups (10 rats/group). Carcinogen-treated rats were administered intragastrically 4 NMBA doses (2 mg/kg body weight), once a week for 4 consecutive weeks. NMBA-untreated groups received saline. The animals were weighed weekly and monitored daily. The study was concluded at 17 weeks after the 1st NMBA dose (22 weeks of ZD). At sacrifice, the animals were anesthetized by delivering isoflurane (GE Healthcare) to the respiratory tract of the rat using a vaporizer at 3% concentration. Blood was obtained from the retro-orbital venous plexus for serum preparation and subsequent Zn analysis. Whole esophagus was excised and longitudinally slit open. Tumors greater than 0.5 mm in diameter were mapped.

### Esophageal epithelia preparation

Esophagi were isolated and cut into two equal portions. Esophageal epithelium was prepared from a portion by using a blade to remove the submucosal and muscularis layers, snap-frozen in liquid nitrogen and stored at −80°C [13]. The remaining portion was fixed in 10% buffered formalin and paraffin embedded.

### RNA isolation

Esophageal epithelial samples frozen in liquid nitrogen were pulverized to a fine powder using a chilled hammer. Total RNA was extracted from the pulverized samples using an animal tissue RNA extraction Kit (#25700, Norgen Biotek, Ontario, Canada). RNA concentration of each sample was determined using a NanoDrop 1000 (Thermo Scientific). All RNA samples displayed a 260:280 ratio >1.8, and a 260:230 ratio > 1.8.

### nanoString rat miRNA expression assay

The nanoString rat miRNA expression assay kit that profiles 423 rat miRNAs was employed (*n* = 6 rats/group). This assay was performed at the Ohio State University Comprehensive Cancer Center Genomics Shared Resource according to manufacturer's instruction. Briefly, 100 ng of total RNA was used as input material. Small RNA samples were prepared by ligating a specific DNA tag onto the 3′ end of each mature miRNA. These tags normalized the melting temperatures (Tms) of the miRNAs and provided identification for each miRNA species in the sample. Excess tags were then removed, and the resulting material was hybridized with a panel of miRNA:tag-specific nCounter capture and barcoded reporter probes. Hybridization reactions were incubated at 64°C for 18 h. Hybridized probes were purified and immobilized on a streptavidin-coated cartridge using the nCounter Prep Station. nCounter Digital Analyzer was used to count individual fluorescent barcodes and quantify target RNA molecules present in each sample. For each assay, a high-density scan (600 fields of view) was performed.

### nanoString data analysis

Abundances of miRNAs were quantified using the nanoString nCounter gene expression system [56]. Each sample was normalized using the global sum method that uses the entire miRNA content. The nanoString nSolver software tool was used to facilitate normalization. Student's t-test was used to calculate statistical significances of pair-wise comparisons. Calculations were performed using the R statistical computing environment (http://www.r-project.org/).

### TaqMan miRNA assay

Reverse transcription of miRNAs was performed according to the manufacturer's instructions (Applied Biosystems, Foster City, CA) with a reaction volume of 15 μl containing 350 ng of total RNA. The real-time PCR was performed using the 7300 Real-Time PCR Systems (Applied Biosystems). Each miRNA and endogenous control (snoRNA and U87) was measured in triplicates. As an overall quality control, CT values above 35 were excluded from analysis.

### *In situ* hybridization

miRCURY locked nucleic acid (LNA)™ microRNA detection probes, namely, rno-miR-21, rno-miR-31, rno-miR-223, hsa-miR-31, hsa-miR-223, negative controls (rno-miR-31) with mismatches at two position, were purchased from Exiqon (Vedbaek, Denmark). The oligonucleotides are double DIG-labeled at the 5′- and 3′-ends. ISH was performed on 6 μm FFPE sections as described by Nielsen et al. [91]. Following deparaffinization, rehydration in graded alcohol and proteinase K treatment, tissue sections were hybridized with miR-31 probe (20 nM), miR-223 or miR-21 probe (50 nM) in hybridization buffer (Exiqon) at 50°C - 57°C for 14 h in a hybridizer (Dako, Glostrup, Denmark). Following stringent washes in SSC buffers, the sections were blocked against unspecific binding of the detecting antibody, using DIG wash and blocking reagent. miRNA was localized by incubation with 4-nitro-blue tetrazolium (NBT) and 5-brom-4-chloro-3′-indolylphosphate (BCIP) (Roche, Mannheim, Germany). Nuclear fast red (Vector Lab., Burlingname, CA) was used as a counterstain.

### Quantitative real time PCR

cDNA was reverse transcribed using the High-Capacity cDNA Archive Kit (Applied Biosystems, Foster City, CA) using reverse transcription reaction volumes of 20 μl containing 1 μg of total RNA for each sample according to the manufacturer's protocol. qPCR was performed using pre-designed probes (Applied Biosystems), Psmb6 and Oaz1 as the normalizers, and the comparative Ct method.

### Immunohistochemistry (IHC)

Formalin-fixed, paraffin-embedded (FFPE) tissues were deparaffinized, and rehydrated in graded alcohols. IHC was carried out as previously described [37, 38, 60] using primary antibodies for PCNA (clone PC-10, Ab-1, Thermo Scientific, Waltham, MA, USA), KRT14 (NCL-LL002, Novocastra, Buffalo Grove, IL, USA), COX-2 (#12282, Cell Signaling, Danvers, MA, USA), S100A8 (T-1032, BMA, Augst, Switzerland), PDCD4 (LS-B1388, Lifespan Biosciences, Seattle, WA), STK40 (orb101780, Biorbyt, Cambridge, United Kingdom), FBXW7 (ab109617, Abcam, Cambridge, MA, USA), and PTEN (#9188, Cell Signaling) after citrate-based antigen retrieval. Protein was localized by incubation with 3-amino-9-ethylcarbazole substrate-chromogen (Dako, Carpinteria, CA, USA) or 3,3′-diaminobenzidine tetrahydrochloride (Sigma-Aldrich, St Louis, MO, USA).

### Microscopy

IHC and ISH analyses were performed by light microscopy using an Olympus BX51 microscope and photographs taken with a Spot RT3 camera and Spot software v. 4.6.

### Zn measurement

Serum Zn content was determined using Atomic Absorption Spectrometer Analyst 400 (PerkinElmer, Waltham, MA).

### Statistical analysis

Dietary Zn effects on continuous data (tumor multiplicity, serum Zn, weight) were analyzed by analysis of variance (ANOVA). Differences among the groups were assessed using the Tukey-HSD multiple comparisons post hoc t-tests. When the data exhibited heteroscedasticity (tested by Levene's homogeneity of variance test), the Welch ANOVA test was used to detect an overall difference in the dietary groups and the Games-Howell pairwise comparison test was used for detecting differences among the groups. For data where only 2 groups were analyzed or for the inflammation genes where we were only interested in detecting differences between the zinc sufficient group and each zinc deficient group, the students t-test was used to compare the groups. Dietary group effects in tumor/ESCC incidence were assessed by an overall chi-square test. Pairwise Fisher's exact test was used to compare the individual dietary groups. Statistical tests were 2-sided and were considered significant at *P* < 0.05. Statistical analysis was performed using R (http://www.R-project.org).

## SUPPLEMENTARY FIGURES AND TABLES



## References

[1] Ferlay J, Soerjomataram I, Dikshit R, Eser S, Mathers C, Rebelo M, Parkin DM, Forman D, Bray F (2015). Cancer incidence and mortality worldwide: sources, methods and major patterns in GLOBOCAN 2012. Int J Cancer.

[2] Arnold M, Soerjomataram I, Ferlay J, Forman D (2015). Global incidence of oesophageal cancer by histological subtype in 2012. Gut.

[3] Magee PN (1989). The experimental basis for the role of nitroso compounds in human cancer. Cancer Surv.

[4] Abnet CC, Lai B, Qiao YL, Vogt S, Luo XM, Taylor PR, Dong ZW, Mark SD, Dawsey SM (2005). Zinc concentration in esophageal biopsy specimens measured by x-ray fluorescence and esophageal cancer risk. Journal of the National Cancer Institute.

[5] Kmet J, Mahboubi E (1972). Esophageal cancer in the Caspian littoral of Iran: initial studies. Science.

[6] Yang CS (1980). Research on esophageal cancer in China: a review. Cancer Res.

[7] Hashemian M, Poustchi H, Abnet CC, Boffetta P, Dawsey SM, Brennan PJ, Pharoah P, Etemadi A, Kamangar F, Sharafkhah M, Hekmatdoost A, Malekzadeh R (2015). Dietary intake of minerals and risk of esophageal squamous cell carcinoma: results from the Golestan Cohort Study. Am J Clin Nutr.

[8] Dar NA, Mir MM, Salam I, Malik MA, Gulzar GM, Yatoo GN, Ahmad A, Shah A (2008). Association between copper excess, zinc deficiency, and TP53 mutations in esophageal squamous cell carcinoma from Kashmir Valley, India—a high risk area. Nutr Cancer.

[9] Lee DH, Anderson KE, Folsom AR, Jacobs DR (2005). Heme iron, zinc and upper digestive tract cancer: the Iowa Women's Health Study. Int J Cancer.

[10] McClain CJ, Su LC (1983). Zinc deficiency in the alcoholic: a review. Alcohol Clin Exp Res.

[11] Abnet CC, Lai B, Qiao YL, Vogt S, Luo XM, Taylor PR, Dong ZW, Mark SD, Dawsey SM (2005). Zinc concentration in esophageal biopsy specimens measured by x-ray fluorescence and esophageal cancer risk. J Natl Cancer Inst.

[12] Taccioli C, Wan SG, Liu CG, Alder H, Volinia S, Farber JL, Croce CM, Fong LY (2009). Zinc replenishment reverses overexpression of the proinflammatory mediator S100A8 and esophageal preneoplasia in the rat. Gastroenterology.

[13] Taccioli C, Chen H, Jiang Y, Liu XP, Huang K, Smalley KJ, Farber JL, Croce CM, Fong LY (2012). Dietary zinc deficiency fuels esophageal cancer development by inducing a distinct inflammatory signature. Oncogene.

[14] Alder H, Taccioli C, Chen H, Jiang Y, Smalley KJ, Fadda P, Ozer HG, Huebner K, Farber JL, Croce CM, Fong LY (2012). Dysregulation of miR-31 and miR-21 induced by zinc deficiency promotes esophageal cancer. Carcinogenesis.

[15] Lin RJ, Xiao DW, Liao LD, Chen T, Xie ZF, Huang WZ, Wang WS, Jiang TF, Wu BL, Li EM, Xu LY (2012). MiR-142-3p as a potential prognostic biomarker for esophageal squamous cell carcinoma. J Surg Oncol.

[16] Zhang T, Wang Q, Zhao D, Cui Y, Cao B, Guo L, Lu SH (2011). The oncogenetic role of microRNA-31 as a potential biomarker in oesophageal squamous cell carcinoma. Clin Sci (Lond).

[17] Ambros V (2003). MicroRNA pathways in flies and worms: growth, death, fat, stress, and timing. Cell.

[18] He L, Thomson JM, Hemann MT, Hernando-Monge E, Mu D, Goodson S, Powers S, Cordon-Cardo C, Lowe SW, Hannon GJ, Hammond SM (2005). A microRNA polycistron as a potential human oncogene. Nature.

[19] Lu J, Getz G, Miska EA, Alvarez-Saavedra E, Lamb J, Peck D, Sweet-Cordero A, Ebert BL, Mak RH, Ferrando AA, Downing JR, Jacks T, Horvitz HR (2005). MicroRNA expression profiles classify human cancers. Nature.

[20] Zeitels LR, Acharya A, Shi G, Chivukula D, Chivukula RR, Anandam JL, Abdelnaby AA, Balch GC, Mansour JC, Yopp AC, Richardson JA, Mendell JT (2014). Tumor suppression by miR-26 overrides potential oncogenic activity in intestinal tumorigenesis. Genes Dev.

[21] Calin GA, Croce CM (2006). MicroRNA signatures in human cancers. Nat Rev Cancer.

[22] Mathe EA, Nguyen GH, Bowman ED, Zhao Y, Budhu A, Schetter AJ, Braun R, Reimers M, Kumamoto K, Hughes D, Altorki NK, Casson AG, Liu CG (2009). MicroRNA expression in squamous cell carcinoma and adenocarcinoma of the esophagus: associations with survival. Clin Cancer Res.

[23] Komatsu S, Ichikawa D, Takeshita H, Tsujiura M, Morimura R, Nagata H, Kosuga T, Iitaka D, Konishi H, Shiozaki A, Fujiwara H, Okamoto K, Otsuji E (2011). Circulating microRNAs in plasma of patients with oesophageal squamous cell carcinoma. Br J Cancer.

[24] Feber A, Xi L, Luketich JD, Pennathur A, Landreneau RJ, Wu M, Swanson SJ, Godfrey TE, Litle VR (2008). MicroRNA expression profiles of esophageal cancer. J Thorac Cardiovasc Surg.

[25] Kurashige J, Watanabe M, Iwatsuki M, Kinoshita K, Saito S, Hiyoshi Y, Kamohara H, Baba Y, Mimori K, Baba H (2012). Overexpression of microRNA-223 regulates the ubiquitin ligase FBXW7 in oesophageal squamous cell carcinoma. Br J Cancer.

[26] Wu C, Wang C, Guan X, Liu Y, Li D, Zhou X, Zhang Y, Chen X, Wang J, Zen K, Zhang CY, Zhang C (2014). Diagnostic and prognostic implications of a serum miRNA panel in oesophageal squamous cell carcinoma. PLoS One.

[27] Tanaka K, Miyata H, Sugimura K, Fukuda S, Kanemura T, Yamashita K, Miyazaki Y, Takahashi T, Kurokawa Y, Yamasaki M, Wada H, Nakajima K, Takiguchi S (2015). miR-27 is associated with chemoresistance in esophageal cancer through transformation of normal fibroblasts to cancer-associated fibroblasts. Carcinogenesis.

[28] Ren LH, Chen WX, Li S, He XY, Zhang ZM, Li M, Cao RS, Hao B, Zhang HJ, Qiu HQ, Shi RH (2014). MicroRNA-183 promotes proliferation and invasion in oesophageal squamous cell carcinoma by targeting programmed cell death 4. Br J Cancer.

[29] Zhao Y, Schetter AJ, Yang GB, Nguyen G, Mathe EA, Li P, Cai H, Yu L, Liu F, Hang D, Yang H, Wang XW, Ke Y (2013). microRNA and inflammatory gene expression as prognostic marker for overall survival in esophageal squamous cell carcinoma. Int J Cancer.

[30] Liu SG, Qin XG, Zhao BS, Qi B, Yao WJ, Wang TY, Li HC, Wu XN (2013). Differential expression of miRNAs in esophageal cancer tissue. Oncol Lett.

[31] Stanitz E, Juhasz K, Gombos K, Gocze K, Toth C, Kiss I (2015). Alteration of miRNA expression correlates with lifestyle, social and environmental determinants in esophageal carcinoma. Anticancer Res.

[32] Yu T, Cao R, Li S, Fu M, Ren L, Chen W, Zhu H, Zhan Q, Shi R (2015). MiR-130b plays an oncogenic role by repressing PTEN expression in esophageal squamous cell carcinoma cells. BMC Cancer.

[33] Zhu L, Yan W, Rodriguez-Canales J, Rosenberg AM, Hu N, Goldstein AM, Taylor PR, Erickson HS, Emmert-Buck MR, Tangrea MA (2011). MicroRNA analysis of microdissected normal squamous esophageal epithelium and tumor cells. Am J Cancer Res.

[34] Zhang C, Wang C, Chen X, Yang C, Li K, Wang J, Dai J, Hu Z, Zhou X, Chen L, Zhang Y, Li Y, Qiu H (2010). Expression profile of microRNAs in serum: a fingerprint for esophageal squamous cell carcinoma. Clin Chem.

[35] Dong W, Li B, Wang Z, Zhang Z, Wang J (2015). Clinical significance of microRNA-24 expression in esophageal squamous cell carcinoma. Neoplasma.

[36] Fong LY, Sivak A, Newberne PM (1978). Zinc deficiency and methylbenzylnitrosamine-induced esophageal cancer in rats. J Natl Cancer Inst.

[37] Fong LY, Zhang L, Jiang Y, Farber JL (2005). Dietary zinc modulation of COX-2 expression and lingual and esophageal carcinogenesis in rats. J Natl Cancer Inst.

[38] Fong LY, Nguyen VT, Farber JL (2001). Esophageal cancer prevention in zinc-deficient rats: rapid induction of apoptosis by replenishing zinc. J Natl Cancer Inst.

[39] Mandard AM, Hainaut P, Hollstein M (2000). Genetic steps in the development of squamous cell carcinoma of the esophagus. Mutat Res.

[40] Iwaya H, Kashiwaya M, Shinoki A, Lee JS, Hayashi K, Hara H, Ishizuka S (2011). Marginal zinc deficiency exacerbates experimental colitis induced by dextran sulfate sodium in rats. J Nutr.

[41] Song Y, Elias V, Loban A, Scrimgeour AG, Ho E (2010). Marginal zinc deficiency increases oxidative DNA damage in the prostate after chronic exercise. Free Radic Biol Med.

[42] Maret W, Sandstead HH (2006). Zinc requirements and the risks and benefits of zinc supplementation. J Trace Elem Med Biol.

[43] Maret W (2013). Zinc and human disease. Metal ions in life sciences.

[44] Kelleher SL, McCormick NH, Velasquez V, Lopez V (2011). Zinc in specialized secretory tissues: roles in the pancreas, prostate, and mammary gland. Advances in nutrition.

[45] Caulfield LE, Black RE, Ezzati M, Lopez AD, Rodgers A, Murray CJ (2004). Zinc deficiency. Comparative Quantification of Health Risk.

[46] Prasad AS (2009). Impact of the discovery of human zinc deficiency on health. J Am Coll Nutr.

[47] Sandstead HH (1991). Zinc deficiency. A public health problem?. Am J Dis Child.

[48] Fong LY, Li JX, Farber JL, Magee PN (1996). Cell proliferation and esophageal carcinogenesis in the zinc-deficient rat. Carcinogenesis.

[49] Dietrich DR (1993). Toxicological and pathological applications of proliferating cell nuclear antigen (PCNA), a novel endogenous marker for cell proliferation. Crit Rev Toxicol.

[50] Cintorino M, Tripod SA, Santopietro R, Antonio P, Lutfi A, Chang F, Syrjanen S, Shen Q, Tosi P, Syrjanen K (2001). Cytokeratin expression patterns as an indicator of tumour progression in oesophageal squamous cell carcinoma. Anticancer Res.

[51] Su H, Hu N, Shih J, Hu Y, Wang QH, Chuang EY, Roth MJ, Wang C, Goldstein AM, Ding T, Dawsey SM, Giffen C, Emmert-Buck MR (2003). Gene expression analysis of esophageal squamous cell carcinoma reveals consistent molecular profiles related to a family history of upper gastrointestinal cancer. Cancer Res.

[52] Liu CG, Zhang L, Jiang Y, Chatterjee D, Croce CM, Huebner K, Fong LY (2005). Modulation of gene expression in precancerous rat esophagus by dietary zinc deficit and replenishment. Cancer Res.

[53] Stoner GD, Gupta A (2001). Etiology and chemoprevention of esophageal squamous cell carcinoma. Carcinogenesis.

[54] Gebhardt C, Nemeth J, Angel P, Hess J (2006). S100A8 and S100A9 in inflammation and cancer. Biochem Pharmacol.

[55] Zimmermann KC, Sarbia M, Weber AA, Borchard F, Gabbert HE, Schror K (1999). Cyclooxygenase-2 expression in human esophageal carcinoma. Cancer Res.

[56] Geiss GK, Bumgarner RE, Birditt B, Dahl T, Dowidar N, Dunaway DL, Fell HP, Ferree S, George RD, Grogan T, James JJ, Maysuria M, Mitton JD (2008). Direct multiplexed measurement of gene expression with color-coded probe pairs. Nat Biotechnol.

[57] Wyman SK, Knouf EC, Parkin RK, Fritz BR, Lin DW, Dennis LM, Krouse MA, Webster PJ, Tewari M (2011). Post-transcriptional generation of miRNA variants by multiple nucleotidyl transferases contributes to miRNA transcriptome complexity. Genome Res.

[58] Guo H, Wang K, Xiong G, Hu H, Wang D, Xu X, Guan X, Yang K, Bai Y (2010). A functional varient in microRNA-146a is associated with risk of esophageal squamous cell carcinoma in Chinese Han. Fam Cancer.

[59] Komatsu S, Ichikawa D, Takeshita H, Tsujiura M, Morimura R, Nagata H, Kosuga T, Iitaka D, Konishi H, Shiozaki A, Fujiwara H, Okamoto K, Otsuji E (2011). Circulating microRNAs in plasma of patients with oesophageal squamous cell carcinoma. Br J Cancer.

[60] Taccioli C, Garofalo M, Chen H, Jiang Y, Tagliazucchi GM, Di Leva G, Alder H, Fadda P, Middleton J, Smalley KJ, Selmi T, Naidu S, Farber JL (2015). Repression of Esophageal Neoplasia and Inflammatory Signaling by Anti-miR-31 Delivery In Vivo. J Natl Cancer Inst.

[61] Xu Y, Sengupta T, Kukreja L, Minella AC (2010). MicroRNA-223 regulates cyclin E activity by modulating expression of F-box and WD-40 domain protein 7. J Biol Chem.

[62] Creighton CJ, Fountain MD, Yu Z, Nagaraja AK, Zhu H, Khan M, Olokpa E, Zariff A, Gunaratne PH, Matzuk MM, Anderson ML (2010). Molecular profiling uncovers a p53-associated role for microRNA-31 in inhibiting the proliferation of serous ovarian carcinomas and other cancers. Cancer Res.

[63] Xu N, Meisgen F, Butler LM, Han G, Wang XJ, Soderberg-Naucler C, Stahle M, Pivarcsi A, Sonkoly E (2013). MicroRNA-31 is overexpressed in psoriasis and modulates inflammatory cytokine and chemokine production in keratinocytes via targeting serine/threonine kinase 40. J Immunol.

[64] Frankel LB, Christoffersen NR, Jacobsen A, Lindow M, Krogh A, Lund AH (2008). Programmed cell death 4 (PDCD4) is an important functional target of the microRNA miR-21 in breast cancer cells. J Biol Chem.

[65] Guan Y, Gorenshteyn D, Burmeister M, Wong AK, Schimenti JC, Handel MA, Bult CJ, Hibbs MA, Troyanskaya OG (2012). Tissue-specific functional networks for prioritizing phenotype and disease genes. PLoS Comput Biol.

[66] Goya J, Wong AK, Yao V, Krishnan A, Homilius M, Troyanskaya OG (2015). FNTM: a server for predicting functional networks of tissues in mouse. Nucleic Acids Res.

[67] Meng F, Henson R, Wehbe-Janek H, Ghoshal K, Jacob ST, Patel T (2007). MicroRNA-21 regulates expression of the PTEN tumor suppressor gene in human hepatocellular cancer. Gastroenterology.

[68] Kim RH, Mak TW (2006). Tumours and tremors: how PTEN regulation underlies both. Br J Cancer.

[69] Tsujimoto Y, Finger LR, Yunis J, Nowell PC, Croce CM (1984). Cloning of the chromosome breakpoint of neoplastic B cells with the t(14;18) chromosome translocation. Science.

[70] Cuilliere-Dartigues P, El-Bchiri J, Krimi A, Buhard O, Fontanges P, Flejou JF, Hamelin R, Duval A (2006). TCF-4 isoforms absent in TCF-4 mutated MSI-H colorectal cancer cells colocalize with nuclear CtBP and repress TCF-4-mediated transcription. Oncogene.

[71] Nakagawa Y, Morikawa H, Hirata I, Shiozaki M, Matsumoto A, Maemura K, Nishikawa T, Niki M, Tanigawa N, Ikegami M, Katsu K, Akao Y (1999). Overexpression of rck/p54, a DEAD box protein, in human colorectal tumours. Br J Cancer.

[72] Akao Y, Marukawa O, Morikawa H, Nakao K, Kamei M, Hachiya T, Tsujimoto Y (1995). The rck/p54 candidate proto-oncogene product is a 54-kilodalton D-E-A-D box protein differentially expressed in human and mouse tissues. Cancer Res.

[73] Goke F, Bode M, Franzen A, Kirsten R, Goltz D, Goke A, Sharma R, Boehm D, Vogel W, Wagner P, Lengerke C, Kristiansen G, Kirfel J (2013). Fibroblast growth factor receptor 1 amplification is a common event in squamous cell carcinoma of the head and neck. Mod Pathol.

[74] Weiss J, Sos ML, Seidel D, Peifer M, Zander T, Heuckmann JM, Ullrich RT, Menon R, Maier S, Soltermann A, Moch H, Wagener P, Fischer F (2010). Frequent and focal FGFR1 amplification associates with therapeutically tractable FGFR1 dependency in squamous cell lung cancer. Sci Transl Med.

[75] Kim HS, Lee SE, Bae YS, Kim DJ, Lee CG, Hur J, Chung H, Park JC, Jung da H, Shin SK, Lee SK, Lee YC, Kim HR (2015). Fibroblast growth factor receptor 1 gene amplification is associated with poor survival in patients with resected esophageal squamous cell carcinoma. Oncotarget.

[76] Wang HG, Pathan N, Ethell IM, Krajewski S, Yamaguchi Y, Shibasaki F, McKeon F, Bobo T, Franke TF, Reed JC (1999). Ca2+-induced apoptosis through calcineurin dephosphorylation of BAD. Science.

[77] Iorio MV, Croce CM (2012). MicroRNA dysregulation in cancer: diagnostics, monitoring and therapeutics. A comprehensive review. EMBO Mol Med.

[78] Hanahan D, Weinberg RA (2000). The hallmarks of cancer. Cell.

[79] Fong LY, Nguyen VT, Farber JL, Huebner K, Magee PN (2000). Early deregulation of the the p16ink4a-cyclin D1/cyclin-dependent kinase 4-retinoblastoma pathway in cell proliferation-driven esophageal tumorigenesis in zinc-deficient rats. Cancer Res.

[80] Petrocca F, Visone R, Onelli MR, Shah MH, Nicoloso MS, de Martino I, Iliopoulos D, Pilozzi E, Liu CG, Negrini M, Cavazzini L, Volinia S, Alder H (2008). E2F1-regulated microRNAs impair TGFbeta-dependent cell-cycle arrest and apoptosis in gastric cancer. Cancer Cell.

[81] Gottardo F, Liu CG, Ferracin M, Calin GA, Fassan M, Bassi P, Sevignani C, Byrne D, Negrini M, Pagano F, Gomella LG, Croce CM, Baffa R (2007). Micro-RNA profiling in kidney and bladder cancers. Urol Oncol.

[82] Laios A, O'Toole S, Flavin R, Martin C, Kelly L, Ring M, Finn SP, Barrett C, Loda M, Gleeson N, D'Arcy T, McGuinness E, Sheils O (2008). Potential role of miR-9 and miR-223 in recurrent ovarian cancer. Mol Cancer.

[83] Nakayama KI, Nakayama K (2006). Ubiquitin ligases: cell-cycle control and cancer. Nat Rev Cancer.

[84] Volinia S, Calin GA, Liu CG, Ambs S, Cimmino A, Petrocca F, Visone R, Iorio M, Roldo C, Ferracin M, Prueitt RL, Yanaihara N, Lanza G (2006). A microRNA expression signature of human solid tumors defines cancer gene targets. Proc Natl Acad Sci U S A.

[85] Medina PP, Nolde M, Slack FJ (2010). OncomiR addiction in an in vivo model of microRNA-21-induced pre-B-cell lymphoma. Nature.

[86] Fassan M, Cagol M, Pennelli G, Rizzetto C, Giacomelli L, Battaglia G, Zaninotto G, Ancona E, Ruol A, Rugge M (2010). Programmed cell death 4 protein in esophageal cancer. Oncol Rep.

[87] Wong TS, Liu XB, Wong BY, Ng RW, Yuen AP, Wei WI (2008). Mature miR-184 as Potential Oncogenic microRNA of Squamous Cell Carcinoma of Tongue. Clin Cancer Res.

[88] Lajer CB, Nielsen FC, Friis-Hansen L, Norrild B, Borup R, Garnaes E, Rossing M, Specht L, Therkildsen MH, Nauntofte B, Dabelsteen S, von Buchwald C (2011). Different miRNA signatures of oral and pharyngeal squamous cell carcinomas: a prospective translational study. Br J Cancer.

[89] Bruegger C, Kempf W, Spoerri I, Arnold AW, Itin PH, Burger B (2013). MicroRNA expression differs in cutaneous squamous cell carcinomas and healthy skin of immunocompetent individuals. Exp Dermatol.

[90] Huang J, Teng L, Liu T, Li L, Chen D, Li F, Xu LG, Zhai Z, Shu HB (2003). Identification of a novel serine/threonine kinase that inhibits TNF-induced NF-kappaB activation and p53-induced transcription. Biochem Biophys Res Commun.

[91] Nielsen BS, Jorgensen S, Fog JU, Sokilde R, Christensen IJ, Hansen U, Brunner N, Baker A, Moller S, Nielsen HJ (2011). High levels of microRNA-21 in the stroma of colorectal cancers predict short disease-free survival in stage II colon cancer patients. Clin Exp Metastasis.

