# Supplementary material for: MicroRNA dysregulation and esophageal cancer development depend on the extent of zinc dietary deficiency
Source: Oncotarget. 2016 Feb 21;7(10):10723–38. doi: 10.18632/oncotarget.7561 (PMC4905434; doi:10.18632/oncotarget.7561)
Supplement: Supplementary file 1 [file oncotarget-07-10723-s001.pdf]

MicroRNA dysregulation and esophageal cancer development depend on the extent of zinc dietary deficiency

Supplementary Material

Supplementary Table 1. Esophageal microRNA expression profiling in Zn-modulated rats fed diets containing 3, 6, 12, or 60 mg Zn/kg using the nanoString^{TM} nCounter rat miRNA expression assay kit (n=6 rats/group) (cut off: $P \leq 0.05$, fold-change ≥ 1.3)

ZD3 = marked ZD (zinc-deficiency), 3 mg Zn/kg diet; ZD6 = moderate ZD, 6 mg Zn/kg diet; ZD12 = mild ZD, 12 mg Zn/kg diet; ZS = Zn-sufficiency, 60 mg Zn/kg

** **denotes miRNAs that are similarly up- or downregulated in human esophageal squamous cell carcinoma**

ZD3 vs ZS (22-weeks of dietary regimen)		
**miRNA name**	**Fold-change**	***P*-Value**
**24 Up-regulated**		
**miR-31	4.90	0.0068
**miR-223	4.21	0.0000
**miR-21	4.17	0.0011
**miR-146b	3.93	0.0000
**miR-146a	3.73	0.0000
miR-342-3p	2.94	0.0001
miR-150	2.12	0.0129
miR-425	2.03	0.0005
miR-17-5p	1.94	0.0063
miR-128	1.91	0.0127
miR-132	1.86	0.0262
miR-126	1.78	0.0247
miR-20a+miR-20b-5p	1.76	0.0003
miR-34a	1.69	0.0097
miR-140	1.6	0.0359
miR-10b	1.53	0.0010
miR-3558-3p	1.53	0.0178
let-7i	1.52	0.0309
miR-16	1.47	0.0239
miR-199a-5p	1.45	0.0143
miR-674-5p	1.4	0.0083

ZD6 vs ZS (22-weeks of dietary regimen)		
**miRNA name**	**Fold-change**	***P*-Value**
**18 Up-regulated**		
**miR-146a	1.86	0.0003
miR-342-3p	1.68	0.0037
**miR-31	1.64	0.0267
miR-140	1.6	0.0325
**miR-146b	1.55	0.0362
miR-3558-3p	1.5	0.0271
miR-150	1.47	0.0215
miR-196b	1.47	0.0285
miR-199a-5p	1.47	0.0484
miR-653	1.45	0.0427
miR-295	1.44	0.0220
miR-347	1.43	0.0015
miR-741-3p	1.41	0.0476
miR-98	1.38	0.0007
**miR-27a	1.37	0.0440
miR-29a	1.35	0.0175
miR-181a	1.31	0.0110
miR-30d	1.3	0.0254
**15 Down-regulated**		
miR-301a	0.46	0.0005
miR-204	0.46	0.0052

ZD12 vs ZS (22-weeks of dietary regimen)		
**miRNA name**	**Fold-change**	***P*-Value**
**11 Up-regulated**		
miR-342-3p	1.83	0.0075
**miR-146a	1.78	0.0015
miR-3558-3p	1.65	0.0043
miR-741-3p	1.61	0.0080
miR-150	1.55	0.0017
miR-26a	1.53	0.0223
**miR-31	1.49	0.0232
miR-363	1.42	0.0209
miR-199a-5p	1.41	0.0196
**miR-223	1.39	0.0173
miR-324-5p	1.30	0.0374
**23 Down-regulated**		
miR-224	0.47	0.0244
miR-3557-3p	0.54	0.0240
miR-132	0.56	0.0271
miR-204	0.59	0.0322
miR-375	0.60	0.0485
miR-3577	0.61	0.0356
miR-23b	0.61	0.0001
miR-203	0.62	0.0003
miR-183	0.63	0.0069

miR-322	1.36	0.0447
miR-93	1.34	0.0049
miR-324-5p	1.3	0.0162
**22 Down-regulated**		
miR-204	0.09	0.0005
miR-1224	0.21	0.0078
miR-375	0.28	0.0031
miR-145	0.36	0.0018
miR-224	0.42	0.0053
miR-434	0.49	0.0013
miR-205	0.5	0.0090
miR-328a	0.56	0.0001
miR-200a	0.56	0.0025
miR-143	0.56	0.0136
miR-301a	0.57	0.0203
miR-142-5p	0.59	0.0150
miR-125b-5p	0.61	0.0066
miR-365	0.63	0.0095
miR-99a	0.65	0.0331
miR-23b	0.68	0.0015
miR-3580-5p	0.7	0.0323
miR-200c	0.71	0.0027
miR-222	0.73	0.0495
miR-3557-3p	0.75	0.0457
miR-632	0.75	0.0498
let-7b	0.75	0.0160

miR-1224	0.47	0.0426
miR-132	0.6	0.0351
let-7e	0.63	0.0096
miR-203	0.64	0.0004
miR-221	0.65	0.0007
miR-23b	0.66	0.0001
miR-183	0.66	0.0076
miR-200b	0.67	0.0010
miR-878	0.67	0.0254
miR-130a	0.74	0.0021
miR-25	0.74	0.0025
miR-19b	0.75	0.0068
miR-300-3p	0.75	0.0279

miR-92b	0.64	0.0385
miR-485	0.64	0.0165
miR-3557-5p	0.64	0.0360
miR-429	0.65	0.0332
miR-301a	0.66	0.0227
miR-200b	0.67	0.0004
miR-335	0.68	0.0314
miR-96	0.70	0.0387
miR-221	0.74	0.0081
miR-3563-5p	0.75	0.0283
let-7e	0.75	0.0282
miR-25	0.76	0.0000
miR-200c	0.76	0.0030
miR-23a	0.76	0.0002

Supplementary Table 2. Esophageal microRNA expression profiling of tumor-bearing esophagus from Zn-modulated rats treated with 4 low doses of *N*-nitrosomethylbenzylamine, using the nanoString™ nCounter rat miRNA expression assay kit (n=6 rats/group) (cut off: $P \leq 0.05$, fold-change ≥ 1.3)

T= tumor endpoint; ZD3 = marked ZD (zinc-deficiency), 3 mg Zn/kg diet; ZD6 = moderate ZD, 6 mg Zn/kg diet; ZD12 = mild ZD, 12 mg Zn/kg diet; ZS = Zn-sufficiency, 60 mg Zn/kg

** **denotes miRNAs that are similarly up- or downregulated in human esophageal squamous cell carcinoma**

ZD3T vs ZST (Tumor endpoint)		
**miRNA name**	**Fold-change**	***P*-Value**
**42 Up-regulated**		
**miR-223	3.59	0.0002
**miR-21	3.39	0.0003
**miR-31	3.36	0.0173
**miR-146a	3.07	0.0001
**miR-146b	2.54	0.0041
miR-132	2.43	0.0001
miR-1949	2.36	0.0116
miR-425	2.13	0.0004
**miR-27a	1.98	0.0136
miR-632	1.91	0.0157
miR-429	1.90	0.0014
**miR-221	1.89	0.0017
miR-29a	1.79	0.0015
miR-342-3p	1.72	0.0095
**miR-27b	1.72	0.0085
**miR-194	1.69	0.0423
miR-96	1.68	0.0145
**miR-24	1.67	0.0061
let-7i	1.66	0.0010
**miR-203	1.65	0.0063
miR-20a+miR-20b-5p	1.65	0.0265
**miR-183	1.64	0.0083
miR-17-5p	1.61	0.0485
miR-378	1.60	0.0101
**miR-130b	1.59	0.0130
miR-93	1.59	0.0001
miR-30a	1.57	0.0192
miR-340-3p	1.57	0.0196

ZD6T vs ZST (Tumor endpoint)		
**miRNA name**	**Fold-change**	***P*-Value**
**23 Up-regulated**		
miR-3575	2.59	0.0205
**miR-223	2.19	0.0111
miR-375	1.97	0.0387
miR-17-2-3p	1.84	0.0209
miR-3596d	1.78	0.0339
miR-29a	1.75	0.0043
miR-30c	1.74	0.0140
miR-504	1.70	0.0012
miR-345-5p	1.69	0.0126
miR-7a	1.65	0.0119
**miR-31	1.62	0.0393
miR-32	1.59	0.0130
miR-411	1.59	0.0108
miR-410	1.58	0.0436
miR-98	1.55	0.0460
**miR-27b	1.54	0.0276
let-7i	1.50	0.0055
miR-3590-3p	1.49	0.0362
miR-188	1.46	0.0120
let-7a	1.46	0.0374
miR-543	1.44	0.0394
miR-300-3p	1.35	0.0336
miR-200b	1.30	0.0334

ZD12T vs ZST (Tumor endpoint)		
**miRNA name**	**Fold-change**	***P*-Value**
**17 Up-regulated**		
**miR-223	2.85	0.0496
miR-3575	2.47	0.0038
miR-1949	2.41	0.0025
miR-7a	2.10	0.0070
miR-98	1.89	0.0005
miR-425	1.89	0.0168
miR-741-3p	1.86	0.0418
miR-26a	1.84	0.0087
miR-671	1.80	0.0381
**miR-31	1.71	0.0468
miR-3596d	1.63	0.0432
miR-543	1.59	0.0049
miR-185	1.52	0.0467
let-7i	1.46	0.0279
miR-107	1.45	0.0040
miR-361	1.37	0.0126
miR-675	1.37	0.0499
**1 Down-regulated**		
miR-205	0.50	0.0215

**miR-107**	1.56	0.0199
**miR-543**	1.56	0.0471
**miR-300-3p**	1.53	0.0031
**miR-148b-3p**	1.52	0.0004
**miR-30c**	1.46	0.0292
**let-7f**	1.46	0.0387
****miR-106b**	1.44	0.0196
****miR-22**	1.41	0.0454
**miR-542-3p**	1.40	0.0360
**miR-295**	1.40	0.0314
**miR-30b-5p**	1.38	0.0306
**miR-411**	1.37	0.0373
**miR-15b**	1.36	0.0468
**miR-191**	1.34	0.0124
**6 Down-regulated**		
**miR-1224**	0.30	0.0498
**miR-224**	0.44	0.0164
**miR-434**	0.46	0.0253
**miR-204**	0.56	0.0361
**miR-139-3p**	0.60	0.0485
**miR-349**	0.63	0.0013
